# Distracted to a fault: Attention, actions, and time perception

**DOI:** 10.3758/s13414-022-02632-x

**Published:** 2022-12-15

**Authors:** Katharina A. Schwarz, Lisa Weller

**Affiliations:** Institute of Psychology, University of Würzburg, Röntgenring 11, 97070 Würzburg, Germany

**Keywords:** Attention, Perception and action, Temporal processing, Temporal binding

## Abstract

In the last years, it has become general consensus that actions change our time perception. Performing an action to elicit a specific event seems to lead to a systematic underestimation of the interval between action and effect, a phenomenon termed temporal (or previously intentional) binding. Temporal binding has been closely associated with sense of agency, our perceived control over our actions and our environment, and because of its robust behavioral effects has indeed been widely utilized as an implicit correlate of sense of agency. The most robust and clear temporal binding effects are typically found via Libet clock paradigms. In the present study, we investigate a crucial methodological confound in these paradigms that provides an alternative explanation for temporal binding effects: a redirection of attentional resources in two-event sequences (as in classical operant conditions) versus singular events (as in classical baseline conditions). Our results indicate that binding effects in Libet clock paradigms may be based to a large degree on such attentional processes, irrespective of intention or action-effect sequences. Thus, these findings challenge many of the previously drawn conclusions and interpretations with regard to actions and time perception.

## Introduction

Acting changes our time perception. When we perform a simple action (e.g., pressing a key) to elicit a subsequent effect (e.g., a tone) after a short delay, performing this action-effect sequence seems to lead to an underestimation of the time interval in between action and effect (Haggard et al., 2002; Muth et al., 2022; Ruess et al., 2017, 2018; Schwarz, Weller, Pfister, & Kunde, 2019b; Tanaka et al., 2019; Tramacere & Allen, 2022). We thus likely perceive action and effect as temporally closer together than they actually are, and we estimate actions and effects differently in time as part of an action-effect sequence than when we encounter them individually (i.e., pressing only a key without consequence or hearing a tone without producing it ourselves). Because this effect seemed to verge on acting *intentionally*, it was originally termed *intentional binding* (Antusch et al., 2019; Haggard et al., 2002). Over the course of several years, intentional binding has become a prolific paradigm in cognitive psychology, the more so, because it has been broadcast as an implicit indicator of sense of agency, the subjective perception of control for our actions and their consequences (Haggard, 2017; Haggard & Tsakiris, 2009; Hoerl et al., 2020; Moore, 2016; Moore et al., 2010; Synofzik et al., 2013; Venskus et al., 2021). Because sense of agency has wide implications for a broad number of subjects, the association of intentional binding and sense of agency has dramatically increased the prominence of the intentional binding paradigm in experimental, cognitive psychology.

This interest in turn has led to an increasing number of studies focusing on intentional binding itself. Assumptions that have gone largely unchallenged over years have now been closely inspected and tested, resulting in often controversial and surprising findings. For example, studies found that intention is indeed not necessary for intentional binding to occur, stimulating a renaming attempt to the more accurate term *temporal binding* (Kirsch et al., 2019; Suzuki et al., 2019). The association of temporal binding and sense of agency is also not as clear as previously assumed, on an experimental as well as on a conceptual level (Majchrowicz & Wierzchoń, 2018; Schwarz, Weller, Klaffehn, & Pfister, 2019a). Studies have found little evidence for correlations between sense of agency measures and temporal binding (Antusch et al., 2021; Saito et al., 2015; Schwarz, Weller, Klaffehn, & Pfister, 2019a; but see Imaizumi & Tanno, 2019), and have sought to find underlying mechanisms for temporal binding spanning from causality perception to multisensory integration that are indeed not synonymous with sense of agency (Antusch et al., 2020; Hoerl et al., 2020; Klaffehn et al., 2021). Moreover, the measuring of temporal binding has come under scrutiny with neither different measures of temporal binding nor subcomponents of the same temporal binding measure relating to one another (Siebertz & Jansen, 2022; Tonn et al., 2021). Conceptually, temporal binding has been questioned as being a measure of perception at all versus a phenomenon based on judgment procedures (Ivanof et al., 2021; Reddy, 2021).

Temporal binding is usually measured by means of two popular methods. In the Libet clock paradigm, participants are asked to monitor a running clock hand while performing a simple action to elicit a subsequent effect, thereby using the clock hand to estimate the point in time of the action or of the effect. Such conditions are termed operant, as they include both an action and the effect elicited by that action. These operant conditions are then calculated against baseline estimations, in which action and effect (i.e., the stimulus that constitutes the effect in the operant condition) are isolated occurrences and no action-effect sequence is present. Thus, in baseline conditions, participants only perform the action and estimate the timing of its occurrence, or they only perceive the effect (i.e., the stimulus that constitutes the effect in the operant condition) without producing it themselves and estimating the timing of its presentation. Such baseline conditions are used to control for biases based on the estimation and monitoring procedure itself, irrespective of the underlying action-effect sequence.

In this paradigm, actions in action-effect sequences are usually perceived as later than actions in baseline conditions, i.e., they are bound towards the effect (action binding), and effects in action-effect sequences are usually perceived as earlier than the same stimuli in baseline conditions, i.e., they are bound towards the action (effect binding). Action and especially effect binding are very robust and stable across participants, and have been corroborated in many studies (Tanaka et al., 2019). A second method of measuring temporal binding is through interval estimations. In these studies, participants are asked to directly estimate the time interval between actions and their effects. Tendentially, participants estimate time intervals to be shorter for action-effect sequences than for two other events (Dewey & Knoblich, 2014). However, findings in these studies are less robust and less clear in their conclusions than in the Libet clock paradigm (Siebertz & Jansen, 2022; Tanaka et al., 2019).

**Fig. 1:**
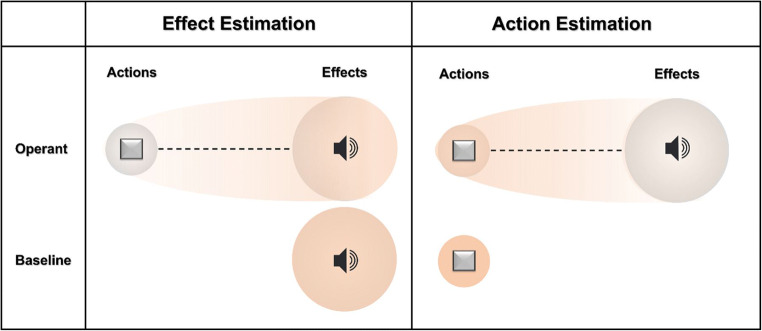
Model of a potential distraction of attentional resources in operant and baseline conditions. The left side represents effect estimations, the right side action estimations. Rectangles symbolize the events (action/effects), grey circles mimic the difference in perceptual certainty for both event types with actions associated with higher perceptual certainty than effects, likely explaining the typically bigger effect sizes for effect binding than for action binding. Rose-colored circles represent attentional resources available for the estimation task. Concentration of the attentional resource is illustrated by the intensity of color. Operant and baseline conditions differ in the number of events they offer in sequence. Operant conditions include both action and effect, potentially distracting attentional resources away from the event that needs to be monitored for estimation purposes towards the respective other event. This could bias time estimations towards the other event (e.g., due to facilitated partial multisensory integration or estimating heuristics), leading to similar result patterns as seen in typical temporal binding paradigms. Baseline conditions only feature one event at a time, thereby bundling attentional resources on the event that needs to be monitored, potentially dependent on the participants’ perceptual certainty

In the present study, we aim to identify and address a decisive, methodologic problem in measuring temporal binding. In the Libet clock paradigm (Haggard et al., 2002; Libet et al., 1983), we constantly compare two conditions, the operant and baseline conditions, and differences between time estimations between these conditions are interpreted as indicators for temporal integration of action and effect in action-effect sequences.^1^ However, baseline and operant conditions differ in a crucial detail beyond the presence or absence of such action-effect sequences: baseline conditions feature one event to draw attentional resources, whereas operant conditions feature two events that can both draw attentional resources. Indeed, typical action-effect sequences draw sustained attention, from attentional orienting over performance monitoring and, specifically, effect monitoring, even if effects are irrelevant (Kok et al., 2006; Schaaf et al., 2022; Wirth et al., 2018). In contrast, singular events, i.e., actions without subsequent effects (aside from proprioceptive feedback) or stimulus appearance without preceding actions, represent shorter time frames and/or lack some of the attention-drawing features of a combined action-effect or two-event sequence. This suggests that the binding effect we see in temporal binding paradigms could to some degree, or even entirely, be due to the distraction of attentional resources away from the event that needs to be monitored for time estimation and towards the other event. Such an effect would lead to precisely the same predictions regarding results as the assumption of skewed time perception due to acting: in operant conditions, actions should be bound towards effects, and effects towards actions, simply due to this distraction of attentional resources (see Fig. 1). This distraction of attentional resources, in turn, could facilitate partial multisensory integration (e.g., Debats et al., 2017; Klaffehn et al., 2021) leading to a higher perceptual overlap of both events. For example, it is quite possible that focus of attentional resources may change perceptual certainty of either event, thus affecting multisensory integration. Alternatively, due to their distraction, participants might be more inclined to follow simple heuristics in their time estimations rather than real perceptual changes, tending towards a “middle ground” in between both events (e.g., Reddy, 2021).

To test our hypothesis, we thus implemented a novel, experimental paradigm in combination with the typical baseline and operant conditions in the Libet clock paradigm. That is, as in classic temporal binding experiments, participants had to press a key and thus elicit a tone in the operant condition (*Operant Classic, OC*) and were then asked to estimate either the timing of the key-press or of the tone presentation (action and effect blocks). In respective baseline conditions (*Baseline Classic, BC*), participants also either had to press a key and estimate the time of key-press (action block) or listen to a tone and estimate the time of tone presentation (effect block).

Moreover, we included two novel conditions to evaluate whether binding effects are based on action-effect sequences or on the distraction of attentional resources due to a two-event sequence. In the *Baseline Attention* (*BA*) task, participants either had to press a key and estimate the time of key-press (action block) or listened to a tone and estimate the time of tone presentation (effect block), mirroring the *BC* task. However, in addition, participants were told that the Libet clock they monitored for time estimation purposes would change color at some point during the trial and they were to report the color that they witnessed at the end of the trial. This color change could happen at any point, before or after the respective event (key-press/tone). The color change was not causally linked to any action on part of the participant, as was made clear to the participants prior to the experiment. To support this claim, we included a substantial number of seemingly random trials (color changes that appeared long before or long after any event should take place). Thus, the *BA* task includes a second event that possibly distracts attentional resources from the event that needs to be estimated in time (key-press/tone), similar to an action-effect sequence, but without an overt causal relationship between the two events, and without any intentional or predictive qualities due to motor involvement. Of course, we cannot preclude that participants still felt a causal relation between both events in this paradigm, and, indeed, if they did, this does not affect our main question: Is temporal binding a measure of skewed time perception due to acting or can it be elicited by any attention-capturing two-event sequence, irrespective of action-effect processes?

Finally, we also included a control condition for the experimental *BA* task, in which participants were to report a colored stimulus at the end of the trial, but this stimulus always (and known to the participants) appeared at the beginning of the trial prior to any events (key-press/tone). This *Baseline Memory (BM)* task was intended to control for the additional effort and attentional resources needed to perform the secondary task (color identification, memorization throughout the task, and reporting at the end of the trial), as well as for biases based on the estimation and monitoring process alone.

Thus, altogether, we employed four different tasks: two experimental (i.e., two-event) tasks, Operant Classic and Baseline Attention, and their respective control (i.e., one-event) tasks, Baseline Classic and Baseline Memory. We expected that distracting attention away from the event participants are asked to estimate in time will evoke temporal binding effects comparable to classic operant conditions. We further hypothesized that the direction of event mis-estimation in the *Baseline Attention* task will be directly related to the temporal occurrence of the color change, also leading to binding effects not seen in classic operant tasks, such as actions being perceived as earlier when the color change occurs prior to the key-press or effects being perceived as later when the color change occurs after the tone presentation. Thus, even in these circumstances, two events would be temporally bound together (color change and action or effect and color change), rendering them instances of temporal binding. However, as such effects would be opposite to typical temporal binding effects as seen in regular action-effect sequences, we will use the term *reverse binding effect* to describe them accurately. Finally, we assumed that color change will affect the participants’ timing estimations the most if it falls within the usual timing of classic operant conditions.

## Methods

### Participants

We recruited 57 participants (mean age = 29.8 years, range 20–64; 45 females, 12 males, 0 diverse; 54 right-handed, 3 left-handed, 0 ambidextrous). This sample size was based on a power calculation assuming a medium effect size of *d* = 0.5 to ensure a power of 1-β = 0.90, increased by 15% to replace possible drop-outs, resulting in a sample size of ≥ 51. As order of conditions was randomized across participants, we further increased this sample to N = 56 (multiple of 8). An additional participant was recruited due to logistical concerns during the experiment.

Participants gave informed consent prior to the experiment and received monetary compensation for participation. Three participants had to be excluded due to technical difficulties or because they failed to perform the experimental task.

### Apparatus

Participants sat in front of a 24-in. flat screen with a refresh rate of 100 Hz and operated a standard German QWERTZ-keyboard with a number pad. The space bar was used to perform key-presses in the experiment. A pure tone with a frequency of 300 Hz and a duration of 100 ms was used as auditory stimulus presented via headphones. Visual stimuli were presented in white on a black background. The Libet clock used for time estimation had a diameter of about 6 cm and took 2,500 ms for a full rotation. Marks on the clock face indicated 5 “min” on the clock and labels were added for 0, 15, 30, and 45 “min”.

### Procedure

**Fig. 2:**
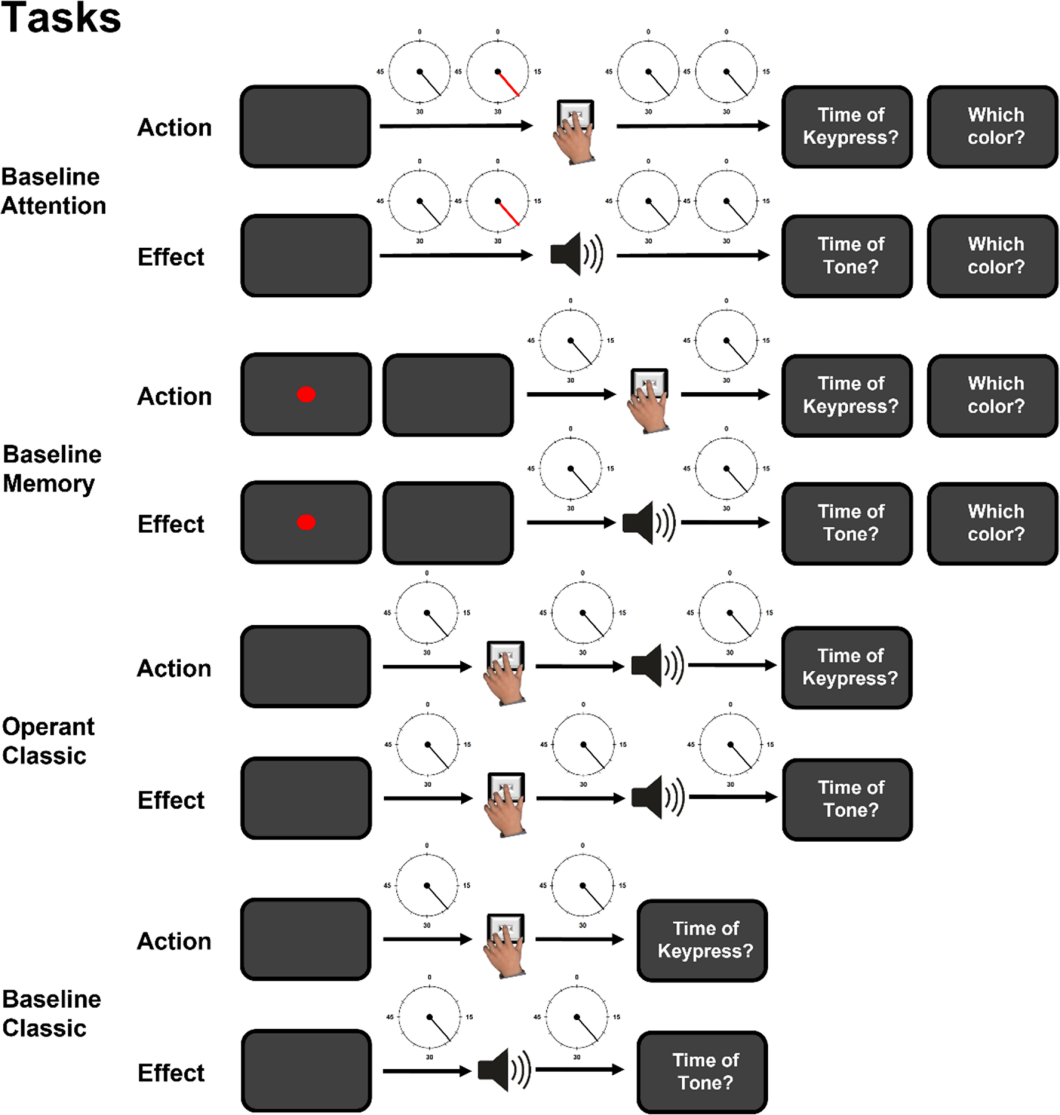
Trial procedure for each condition. In the Baseline Attention (BA) task, participants either pressed a key (action block) or heard a tone (effect block) and were asked to estimate the timing of the respective event via a Libet clock with a rotating clock hand. Sometime during the trial, either before the event (action/effect) or after, the clock hand changed color (blue, green or red) for 200 ms (depicted is a trial in which the color changes to red before the respective event as an example). Participants were asked to report the color they had perceived at the end of the trial. The *Baseline Memory* (BM) task served as a control for the BA task. Here, shortly after the beginning of the trial, a colored circle appeared on the screen for 200 ms. As in the BA task, participants were asked to memorize the color and report it at the end of the trial. After the colored circle vanished, a typical temporal binding baseline trial started, and the participants either pressed a key (action block) or heard a tone (effect block) and were asked to estimate the timing of the respective event (action/effect) via the Libet clock. The *Operant Classic* (OC) task mirrored typical operant conditions of temporal binding paradigms. Participants pressed a key to elicit a tone with a delay of 200 ms, 250 ms, or 300 ms. Participants were then asked to estimate the timing of either the key-press (action block) or the tone (effect block) via the Libet clock. The *Baseline Classic* (BC) task mirrored typical baseline conditions in temporal binding paradigms. Participants either pressed a key (action block) or heard a tone (effect block) and were asked to estimate the timing of the respective event (action/effect) via the Libet clock

The four different tasks (*Operant Classic* (OC), *Baseline Classic* (BC), *Baseline Attention* (BA), and *Baseline Memory* (BM)) were presented in separate blocks. For each task, there was an action and an effect block asking participants to estimate the timing either of their action or of the effect (see Fig. 2), with 36 trials each, except for the BA condition, in which the number of trials was doubled (see below for details).

Each trial in every condition started with a blank screen for 500 ms. Then, the clock appeared with the clock hand at a random position, which immediately started rotating. In the *Operant Classic* condition (OC), participants had to press the key and the tone effect was presented 200 ms, 250 ms, or 300 ms after the key-press (counterbalanced across trials). The clock stopped rotating 2,000–3,000 ms after the key-press and participants were asked to report the timing of one event. In action blocks, participants were asked to estimate the timing of their key-press using the Libet clock; in effect blocks, they were asked to estimate the timing of the tone effect. As in all conditions with key-presses, participants were instructed to press the key after the clock hand had completed half a rotation, but before it had completed a full rotation (i.e., between 1,250 and 2,500 ms after the appearance of the clock). When the key was pressed earlier or later than that, an error message was displayed and the trial was repeated. If participants pressed a key in conditions that did not require a key-press (see below), an error message was displayed and the trial was also repeated.

In all other tasks (i.e., BC, BA, and BM), only one event of the action-effect sequence was presented (key-press or tone effect); otherwise, procedure and timing were similar to the OC task. In the action blocks of the *Baseline Classic* (BC) task, participants had to press a key, but no tone effect was presented. In the BC effect blocks, no key was pressed but the tone was presented 1,250–2,500 ms after the Libet clock started rotating (equivalent to the timing of participants’ key-presses, see above). The BC task served as a control condition to the OC task.

The *Baseline Attention* (BA) task was similar to the BC condition. Additionally, the clock hand changed its color to blue, green, or red for 200 ms within a trial and the participants had to report the color at the end of the trial (following the time estimation). In half of the trials, the color change occurred contingent on the other event within the trial and with the same delay as the delay between action and effect in the OC condition. That is, in BA action blocks, the color change occurred 200 ms, 250 ms, or 300 ms after the participants’ key-press. In BA effect blocks, the color change occurred 200 ms, 250 ms, or 300 ms before the tone was presented. In the other half of the trials, timing of the color change was contingent on the start of the Libet clock (but *not* contingent on the other event in the trial). More precisely, the tone could occur during one of two time periods within a trial, either 750–1,250 ms after the appearance of the clock or 2,500–3,750 ms after appearance of the clock. The two time periods were selected so that the color change would occur clearly before or after the other event (key-press/tone) in the trial. Before the trial, one of the three time periods (very early in the trial, operant time range, very late in the trial) was chosen randomly as was the exact time of color presentation within that time period. This procedure ensured that the color change would occur before the participants’ key-press and after the tone in a substantial number of trials (50%), emphasizing that there was no causal relation between color change and key-press or tone presentation. Participants were told that the color change always occurred at a random time within the trial. Because of this procedure, the number of trials was doubled in the BA conditions compared to the other conditions.

The *Baseline Memory* (BM) task was also similar to the BC task, but featured an additional memory task. To that end, a small colored circle (about 0.5 cm diameter) was presented for 200 ms before the Libet clock appeared. The circle was blue, green, or red, and participants had to report the color at the end of the trial (following the time estimation). The BM task thus served as a control condition for the BA task, featuring similar additional elements such as color identification, memorization, and reporting at the end of the trial.

Action and effect blocks of one task were always presented after one another. The order of conditions was balanced across participants by counterbalancing whether participants started (1) with the classic tasks (OC, BC) or the novel tasks (BA, BM), (2) with a one-event task (BC, BM) or a two-event task (OC, BA), and (3) with the action or the effect block. Participants could take self-paced breaks after each block and the entire experiment lasted about 70 min.

To familiarize participants with the tasks, they completed six practice trials of the OC action condition at the beginning of the experiment and six practice trials of the BA action condition directly before completing the first block of the new conditions (BA or BM).

### Data analysis

Estimation errors were calculated separately for each participant and trial, by subtracting the actual time at the moment of the event in question (key-press or tone) from the estimated time of this event. A negative estimation error therefore indicated that tone or key-press were perceived earlier than they actually occurred, whereas a positive estimation error indicated that tone or key-press were perceived later than they actually appeared. Error trials were excluded (5.4%), as well as trials deviating more than 2.5 standard deviations from the cell mean (2.3%), calculated separately for each participant and condition, i.e., Task (Baseline Classic (BC), Baseline Memory (BM), Baseline Attention (BA), Operant Classic (OC)) and Event (Action, Effect). We further excluded trials with false color reports in the secondary task for the BM and BA task (2.4%).

Temporal binding was calculated by subtracting control conditions from the respective experimental conditions (action binding) and by subtracting the experimental conditions from their control conditions (effect binding), resulting in positive binding scores irrespective of the event that was evaluated (action/effect). This allowed us to analyze differences in the effect size of the participants’ misestimations, irrespective of the direction of misestimation (i.e., earlier or later than the respective control condition). Combined temporal binding scores consisted of the sum of action and effect binding scores. To probe for differences between our experimental conditions, we computed analyses of variances (ANOVAs) with the factors *Task* (BC, BM, BA, OC or OC|BC, BA|BM) and *Event* (Action, Effect). For follow-up analyses, pairwise comparisons were analyzed via two-tailed, paired *t*-tests with corresponding effect sizes being calculated as *d*_*z*_ = *t*/sqrt(n).

We additionally included two control analyses: We compared the Baseline Memory condition with the Baseline Control condition, and we repeated the previous ANOVA with subsequent *t*-tests as follow-up tests by using the BC condition as the control condition for both experimental conditions (i.e., OC|BC, BA|BC).

We further analyzed the influence of delay on binding scores by computing an ANOVA on combined temporal binding scores dependent on delay timing (factors *Task* (OC/BC, BA/BM) and *Delay* (200 ms, 250 ms, 300 ms)).

Finally, we analyzed how binding can be directed by attentional resources. In this analysis, we only targeted the Baseline Attention condition and included all trials in which the color change appeared more than 400 ms^2^ before the event participants were asked to evaluate, all trials in which the color change appeared more than 400 ms after the event participants were asked to evaluate, as well as all trials that fell within the typical range of the operant condition (i.e., less than 400 ms before the event for effect trials or after the event for action trials). We chose to exclude all trials that did not fall within these time frames (i.e., less than 400 ms after the event for effect trials or before the event for action trials) because trial numbers per cell were too small for reliable analyses (3.8% overall). We then calculated separate ANOVAs for action and effect trials with the factor *Direction* (> 400 ms before event vs. operant time range vs. > 400 ms after event) and follow-up *t*-tests.

## Results

While the *Baseline Attention* task featured trials that fell outside the timing parameters of the *Operant Classic* task, for the following analyses, we included only trials similar in timing so that comparisons were not confounded by timing differences (i.e., delays of 200, 250, and 300 ms between key-press and tone; see the *Methods* section for further details).

### Attention and temporal binding: Raw estimates

**Fig. 3:**
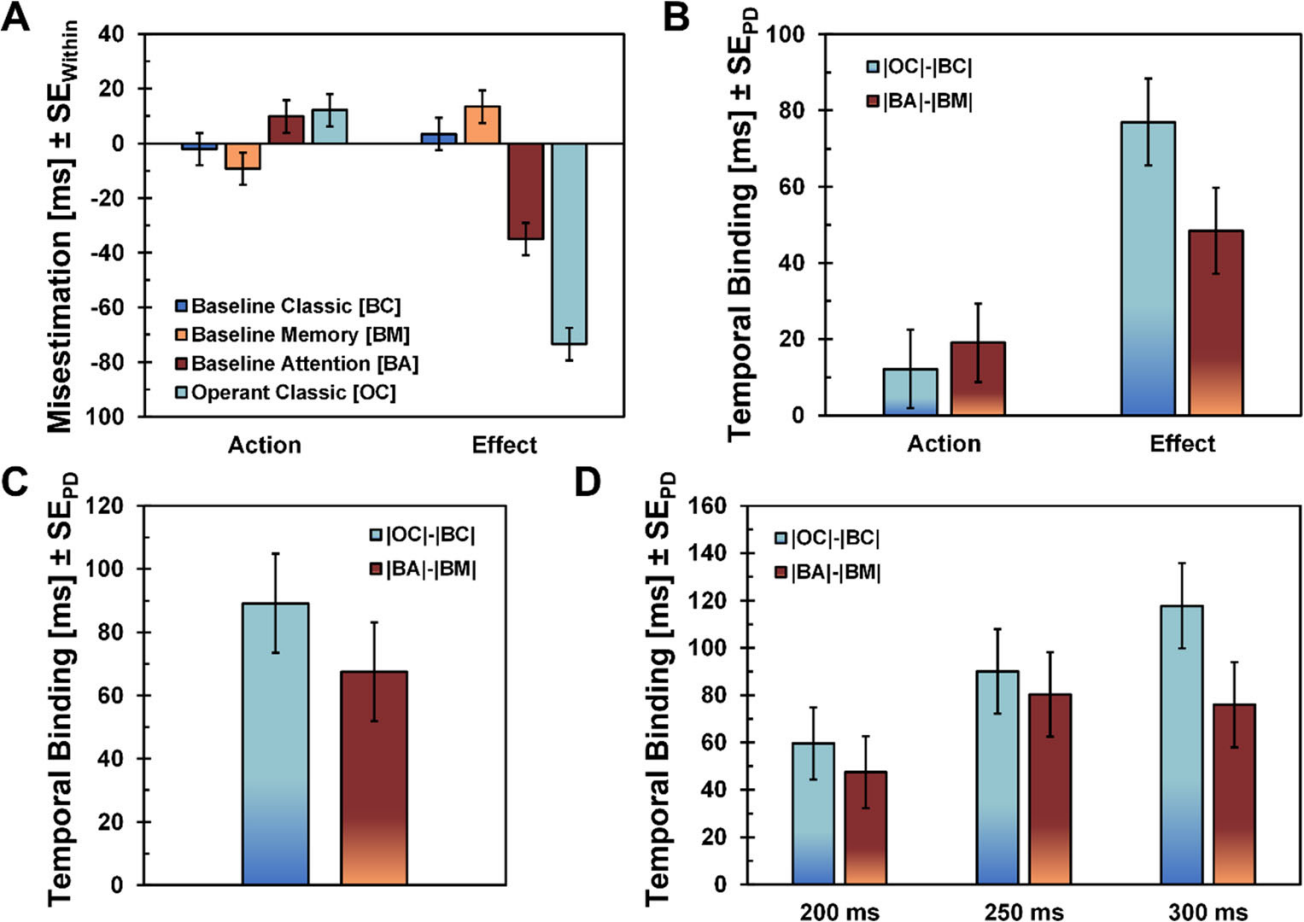
**a** Participants’ misestimations of event timing, separately for each task (*Baseline Classic* (BC), *Baseline Memory* (BM), *Baseline Attention* (BA), and *Operant Classic* (OC)) and event (*Action* (Keypress) and *Effect* (Tone)). Error bars depict within-subjects standard errors, calculated for the main effect *Task* (Loftus & Masson, 1994). **b** Binding scores, calculated by subtracting the respective control condition from the experimental condition (action binding) and by subtracting the experimental condition from the respective control condition (effect binding). **c** Combined temporal binding scores (sum of action and effect binding) for both experimental conditions. Although the operant condition shows descriptively higher binding scores, the difference between both experimental conditions is not significant, *t*(53) = 1.38, *p* = .172, *d*_*z*_ = 0.19. **d** Combined temporal binding scores, separately for each delay condition. A significant difference between both experimental tasks (operant vs. attention) only emerged for the 300-ms delay condition, *t*(53) = 2.32, *p* = .024, *d*_*z*_ = 0.32. For B, C, and D, error bars depict standard errors of the paired difference (Pfister & Janczyk, 2013)

Participants’ misestimations of event timing differed strongly between different experimental tasks, *F*(3,159) = 17.86; *p* < .001, ƞ_p_^2^= .25, ε = 0.75 (GG-corrected), and events, *F*(1,53) = 11.57; *p* = .001, ƞ_p_^2^= .18, with misestimations being most pronounced for effects^3^ in the experimental conditions (*Baseline Attention*, BA, and *Operant Classic*, OC), *F*(3,159) = 41.63; *p* < .001, ƞ_p_^2^= .44, ε = 0.84 (GG-corrected) (see Fig. 3a). Follow-up pairwise comparisons revealed that participants estimated the timing of actions and effects more correctly in control conditions (*Baseline Classic*, BC, and *Baseline Memory*, BM) than in their respective experimental conditions (see Table 1). Interestingly, for actions, misestimations between both experimental conditions (*Operant Classic* and *Baseline Attention*) did not differ significantly, *M*_*OC-BA*_ = 2 ms, *t*(53) = 0.44, *p* =.662, *d*_*z*_ = 0.06, and neither did misestimations between both control conditions (*Baseline Classic* and *Baseline Memory*); *M*_*BC-BM*_ = 7 ms, *t*(53) = 1.63, *p* = .108, *d*_*z*_ = 0.22. For effects, both comparisons did reach significance, with effects being perceived earlier in the *Baseline Classic* task than in the *Baseline Memory* task, *M*_*BC-BM*_ = -10 ms, |*t|*(53) = 2.27, *p* = .027, |*d*_*z*_*|* = 0.31, and earlier in the *Operant Classic* task than in the *Baseline Attention* task, *M*_*OC-BA*_ = -38 ms, |*t|*(53) = 3.61, *p* <.001, |*d*_*z*_*|* = 0.49.

**Table 1:** Means and pairwise comparisons of the participants’ misestimations of actions and effects in all tasks

Task	M_Actions_	Pairwise comparisons (Actions)	M_Effects_	Pairwise comparisons (Effects)
Baseline Classic (BC)	-2 ms	BC vs. OC: *t*(53) = 2.42, *p* =.019, *d*_*z*_ = 0.33 BC vs. BM: *t*(53) = 1.63, *p* = .108, *d*_*z*_ = 0.22	4 ms	BC vs. OC: *t*(53) = 7.62, *p* <.001, *d*_*z*_ = 1.03 BC vs. BM: *t*(53) = 2.27, *p* = .027, *d*_*z*_ = 0.31
Operant Classic (OC)	12 ms	**OC vs. BA:** ***t*(53) = 0.44,** ***p*** **=.662,** ***d***_***z***_ **= 0.06**	-73 ms	**OC vs. BA:** ***t*(53) = 3.61,** ***p*** **<.001,** ***d***_***z***_ **= 0.49**
Baseline Memory (BM)	-9 ms	BM vs. BA: *t*(53) = 4.39, *p* <.001, *d*_*z*_ = 0.60	13 ms	BM vs. BA: *t*(53) = 5.12, *p* < .001, *d*_*z*_ = 0.70
Baseline Attention (BA)	10 ms		-35 ms	

Comparison of the experimental tasks (OC vs. BA) is shown bold

These results indicate that for raw time estimates, action binding could be completely accounted for by attentional processes, whereas the effect size of effect binding was halved when controlling for attentional artefacts introduced by the two-event sequence in operant versus one singular event in baseline conditions.

### Attention and temporal binding: Binding scores

In accordance with classical temporal binding studies, we additionally estimated temporal binding scores by subtracting the control condition (*Baseline Classic*) from the experimental condition (*Operant Classic*) for action binding, and the experimental condition from the control condition for effect binding. This ensures that misestimations are not based on confounds that are introduced into the process by the time estimation procedure alone, irrespective of action-effect sequences. Moreover, we controlled our novel experimental condition, *Baseline Attention*, by performing similar calculations with the control condition *Baseline Memory*. We introduced a special control condition to the experiment to offset confounds that may relate to the memory part of the secondary task, i.e., the memorization and reporting of the perceived color in these conditions (for a closer description of both tasks, see *Methods* section), as well as to the estimation procedure.

When controlled, binding effects no longer differed between the classical operant condition and our novel, experimental condition that introduces an attention-capturing secondary event into a baseline paradigm; main effect *Task*, *F*(1,53) = 1.92, *p* = .172, ƞ_p_^2^= .03 (see Fig. 3b). Binding scores did, however, differ between actions and effects, main effect *Event*, *F*(1,53) = 29.17, *p* < .001, ƞ_p_^2^= .35, with participants showing higher binding for effects compared to actions, mirroring a classical result pattern in temporal binding paradigms (e.g., Schwarz, Weller, Klaffehn, & Pfister, 2019a; Tanaka et al., 2019). Moreover, binding scores differed more strongly between tasks for the effect estimations than for the action estimations, *Task x Event*, *F*(1,53) = 5.61, *p* = .022, ƞ_p_^2^= .10. Indeed, follow-up pairwise comparisons showed that action binding did not differ between controlled operant and baseline attention conditions, *M*_*OC|BC*_ = 12 ms, *M*_*BA|BM*_ = 19 ms, *M*_*Diff*_ = 7 ms, |*t|*(53) = 0.67, *p* = .507, *d*_*z*_ = 0.09. However, effect binding did show a small effect between both tasks, *M*_*OC|BC*_ = 77 ms, *M*_*BA|BM*_ = 48 ms, *M*_*Diff*_ = 29 ms, *t*(53) = 2.52, *p* = .015, *d*_*z*_ = 0.34 (see Fig. 3b). This effect, however, is strongly diminished in comparison to the classical comparison of operant versus baseline (*d*_*z*_ = 1.03), which typically constitutes effect binding. Mirroring the analysis of variance above, a combined temporal binding score for both tasks did not differ significantly, *M*_*Diff*_ = 22 ms, *t*(53) = 1.38, *p* = .172, *d*_*z*_ = 0.19 (see Fig. 3c).

Because the Baseline Memory condition is novel, it is important to be cautious about its use – is it really warranted and necessary? To answer this question, we have included two control analyses targeting the difference between (1) the Baseline Classic and the Baseline Memory condition, and (2) the Baseline Classic and the Baseline Attention condition.

In the Baseline Memory condition there are two additional events compared to the Baseline Classic condition, although both events clearly and predictably fall outside of the actual task, i.e., the timing evaluation of the respective event within this condition (key-press or tone): The first event is the appearance of a colored circle before the beginning of the timing evaluation task and the second event is the color report after the timing evaluation task. Both may potentially draw attention and it is therefore important to see if we find binding effects simply due to these events. For the timing evaluation of actions, we did not find binding effects, |*t|*(53) = 1.63, *p* = .108, |*d*_*z*_| = 0.22; however, this may be due to too little power for such a subtle effect. Interestingly, though, on a purely descriptive basis, binding here was more directed towards the circle appearance prior to the action. In contrast, for the timing evaluation of effects, we found clear evidence for reverse binding towards the color report in the Baseline Memory task, *t*(53) = 2.27, *p* = .027, *d*_*z*_ = 0.31. Thus, this control analysis indicates that the Baseline Memory and Baseline Classic conditions only subtly differ for actions (if at all), but they do differ for effects.

For the sake of transparency, we conducted a second control analysis, that is, we repeated the main temporal binding analysis differentiating between the classic operant and our novel attention condition but used the Baseline Classic condition as a control for both experimental conditions. However, it is important to keep in mind that due to the color report, which also seems to draw attention, results for effect binding are likely an underestimation of the real binding effect. When controlled with the Baseline Classic condition, binding effects differed between the classical operant condition and our novel attention condition; main effect *Task*, *F*(1,53) = 8.07, *p* = .006, ƞ_p_^2^= .13 and they also differed between action and effects, main effect *Event*, *F*(1,53) = 26.31, *p* < .001, ƞ_p_^2^= .33, with participants again showing higher binding for effects compared to actions. As in the original analysis, binding scores differed more strongly between tasks for the effect estimations than for the action estimations, *Task x Event*, *F*(1,53) = 6.78, *p* = .012, ƞ_p_^2^= .11. Follow-up pairwise comparisons showed that action binding did not differ between controlled operant and baseline attention conditions, *M*_*OC|BC*_ = 12 ms, *M*_*BA|BC*_ = 12 ms, *M*_*Diff*_ = <1 ms, *t*(53) = 0.03, *p* = .973, *d*_*z*_ < 0.01. However, effect binding showed an (almost) medium-sized effect between both tasks, *M*_*OC|BC*_ = 77 ms, *M*_*BA|BC*_ = 39 ms, *M*_*Diff*_ = 38 ms, *t*(53) = 3.61, *p* < .001, *d*_*z*_ = 0.49. Despite the potential underestimation of the binding effect as argued above, this effect size is still strongly diminished in relation to the classical comparison (*d*_*z*_ = 1.03 in this experiment). A combined binding score for the control analysis still showed a significant difference between the classical operant condition and our attention condition, *M*_*Diff*_ = 39 ms, *t*(53) = 2.84, *p* = .006, *d*_*z*_ = 0.39, but the effect size is further reduced compared to the analysis of separate binding effects.

Neither of the control analyses change the general results pattern described in this article; however, especially the first control analysis (BM vs. BC) makes it clear that the utilization of a new control condition is warranted and remains the focus of the following analysis.

### The impact of delays

We further analyzed how different delays affected combined temporal binding scores. While the binding scores did not differ between the controlled operant and baseline attention conditions, main effect *Task*, *F*(1,53) = 1.83, *p* = .181, ƞ_p_^2^= .03, the lengths of delays did affect binding scores with higher delays leading to more pronounced temporal binding, main effect *Delay*, *F*(2,106) = 25.87, *p* < .001, ƞ_p_^2^= .33. Moreover, delays seem to have affected binding scores dependent on the task, interaction *Task* x *Delay*, *F*(2,106) = 4.67, *p* = .011, ƞ_p_^2^= .08, an effect that is mainly driven by the comparison of binding scores for the 300-ms delay, *M*_*300,Diff*_ = 42 ms, *t*(53) = 2.32, *p* = .024, *d*_*z*_ = 0.32. Binding scores did not differ between tasks for shorter delays; *250 ms: M*_*250,Diff*_ = 10 ms, *t*(53) = 0.55, *p* = .585, *d*_*z*_ = 0.07; *200 ms*: *M*_*200,Diff*_ = 12 ms, *t*(53) = 0.80, *p* = .428, *d*_*z*_ = 0.11 (see Fig. 3d).

### Does directing attention direct binding?

If attentional processes play a role in temporal binding, color changes should be able to direct participants’ misestimation, no matter whether these color changes appear within the usual action-effect sequence or not. That is, we should be able to elicit temporal misperceptions that fall outside of the usual patterns seen in temporal binding, i.e., actions that are perceived earlier and effects that are perceived later than they actually occurred. In the following analysis, we only analyzed the Baseline Attention condition and included all trials in which the color change appeared long before the event participants were asked to evaluate (> 400 ms before the event) and all trials in which the color change appeared long after the event participants were asked to evaluate (> 400 ms after the event), in addition to the trials that fall within the typical range of the operant condition (i.e., less than 400 ms before the event for effect trials or after the event for action trials).

**Fig. 4:**
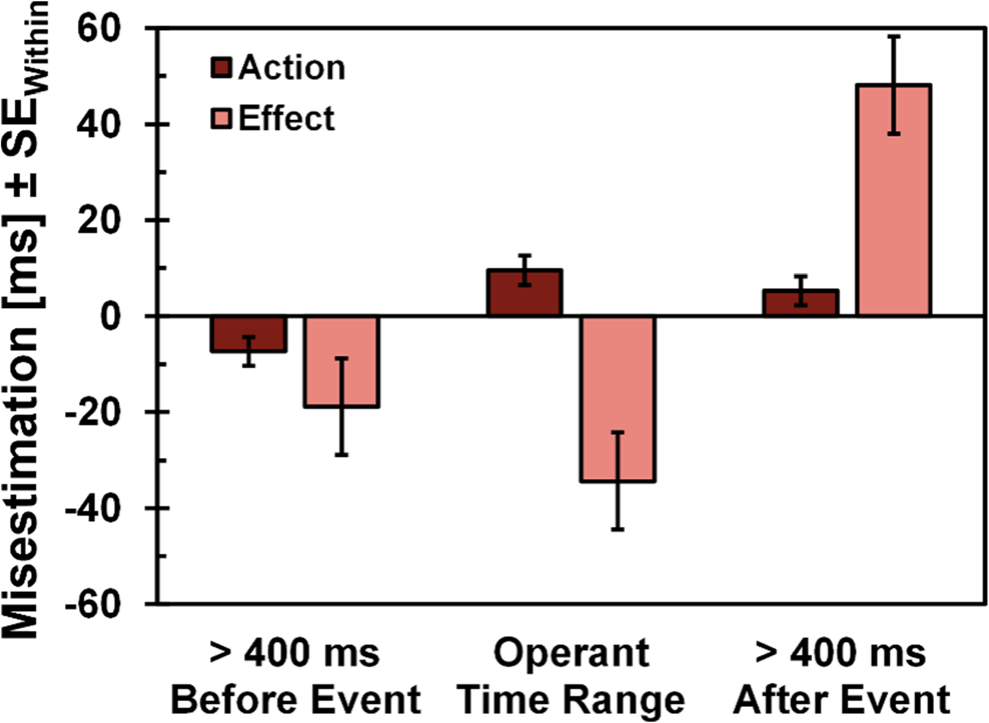
Participants’ estimations of event timing was directed by attention. If color change appeared prior to the event, the event was estimated to occur earlier; if the color change appeared after the event, the event was estimated to occur later. Please note that for action and effect estimations, operant time range was different, i.e., for action estimations, operant time frame included time intervals of 0–400 ms after the event; for effect estimations, operant time frame included time intervals of 0–400 ms prior to the event. Errors bars depict within-subjects standard errors, calculated for the main effect *Direction,* separately for *Action* and *Effect* estimations (Loftus & Masson, 1994)

Participants’ temporal estimations of the presented tones were strongly affected by the timing of the color change (see Fig. 4), *F*(2,106) = 24.05, *p* < .001, ƞ_p_^2^= .31, ε = 0.79 (GG-corrected), with color changes before tone presentation leading to effects being perceived as earlier compared to color changes after tone presentation; *Long After Event* vs. *Long Before Event*, *M*_*LAE*_ = 48 ms, *M*_*LBE*_ = -19 ms, *M*_*Diff*_ = 67 ms, *t*(53) = 4.52, *p* < .001, *d*_*z*_ = 0.61; *Long After Event* vs. *Shortly Before Event (Operant Time Range)*, *M*_*OTR*_ = -34 ms, *M*_*Diff*_ = 82 ms, *t*(53) = 9.24, *p* < .001, *d*_*z*_ = 1.26. However, whether the color change appeared shortly before the event or long before the event did not affect misestimations significantly, although descriptively there seems to be a slightly larger effect for shorter compared to longer delays, *Long Before Event* vs. *Shortly Before Event (Operant Time Range)*, *M*_*Diff*_ = 15 ms, *t*(53) = 1.16, *p* = .252, *d*_*z*_ = 0.16.

The temporal estimations of actions were likewise affected by attention, *F*(2,106) = 11.14, *p* < .001, ƞ_p_^2^= .17, ε = 0.76 (GG-corrected). If color changes appeared before the action, actions were perceived as earlier compared to color changes after the action, *Long After Event* vs. *Long Before Event*, *M*_*LAE*_ = 5 ms, *M*_*LBE*_ = -7 ms, *M*_*Diff*_ = 12 ms, *t*(53) = 3.07, *p* = .002, *d*_*z*_ = 0.42; *Shortly After Event* (*Operant Time Range)* vs. *Long Before Event*, *M*_*OTR*_ = 10 ms, *M*_*Diff*_ = 17 ms, *t*(53) = 3.93, *p* < .001, *d*_*z*_ = 0.53. The impact of the delay length remained only marginally significant, *Long After Event vs. Shortly After Event* (*Operant Time Range), M*_*Diff*_ = 4 ms, *t*(53) = 1.69, *p* = .097, *d*_*z*_ = 0.23, although the difference occurred as expected with shorter delays having a slightly larger influence than longer delays (one-sided *p* = .048). Altogether these results support our hypothesis that attentional resources can predictably direct binding effects and thus also evoke reverse binding.

## Discussion

In the present study, we introduced a simple attentional manipulation in the classic temporal binding paradigm in order to evaluate how binding effects may be the result of the distraction of attentional resources in two-event sequences rather than a phenomenon based on intention, causality, or predictive mechanisms. As typical baseline conditions in Libet clock paradigms only feature one event (i.e., action or effect), whereas typical operant conditions feature two events (i.e., action and effect), this may skew attention towards the event that is not estimated in time, thereby leading to temporal binding effects independent of action-effect sequences.

### Attention and temporal binding

Our results support our hypotheses as we found binding effects for our Baseline Attention task, i.e., a baseline condition featuring only key-press or tone, but including an additional, attention-capturing secondary event, even though there was no action-effect sequence occurring in this condition. Indeed, action binding was similar in size in the Operant Classic and Baseline Attention task. Similarly, effect binding was present in both tasks, even though the effect was larger in the Operant Classic condition. When comparing controlled binding scores, i.e., experimental conditions controlled by their respective control conditions instead of raw estimates, the differences between the two experimental conditions decreased even further, and for combined binding scores, we no longer found significant differences between the classic operant and the novel baseline attention condition. This null effect, however, needs to be considered with caution: whereas the effect size seems reliably decreased in this analysis, statistical significance may well be found at higher sample sizes. Moreover, previous evidence suggests that action and effect binding seem to rely on different mechanisms (Tanaka et al., 2019; Tonn et al., 2021), which renders combined temporal binding effects difficult to interpret despite their common use. Nevertheless, our results indicate that action and effect binding may at least partly be the result of an attentional artefact, irrespective of intention or the presence of an action-effect sequence.

Moreover, our results invite a reinterpretation of previous results: we found effect binding even for deliberate nonactions (although less pronounced than for actions) in a previous study interpreting this finding as intention and causality being sufficient to elicit binding effects even in the absence of actual motor activity (Weller et al., 2020). However, in light of the present results, the decision not to act could have bound attentional resources similarly to actions, albeit to a smaller degree. Likewise, in an opposite study design, we did not find action binding (yet a strong sense of agency) for effect prevention, i.e., in situations in which agents perform an action to prevent the occurrence of a stimulus (Pfister et al., 2021). In accordance with the present study, such an experimental setup would be akin to a singular event with no subsequent event to draw attentional resources which could elicit binding effects.

In fact, the diminished effect size of effect binding scores when controlled for two-event sequences is in a similar range to interval estimation paradigms (Tanaka et al., 2019), potentially supporting the notion that this may be the true effect size of temporal binding that is actually due to action-effect sequences – or causality assumptions, as our experimental design did not differentiate between both explanatory models. A counterargument could, of course, lie in the fact that binding was absent after controlling for two-event sequences only for both shorter delays (200 ms and 250 ms), whereas slightly longer delays (300 ms) showed a (small, but significant) difference. Maybe even longer delays would thus decrease the influence of distraction, and give purer values of temporal binding? While that may be true, it is important to keep in mind that, in fact, temporal binding in Libet clock paradigms typically decreases strongly for longer delays (starting between 250 ms and 400 ms; Ruess et al., 2017), coinciding with the loss of attentional influence, possibly explaining some of the decrease in effect size due to interval length. However, please note that, with the interval estimation procedure, temporal binding effects have also been found for super-second intervals (e.g., Humphreys & Buehner, 2009). Future research should thus look more closely into the interplay of attention and temporal binding for longer action-effect intervals, possibly by introducing longer delays into the experimental paradigm presented in this study.

### Directing temporal binding

Our results further indicate that, as expected, temporal binding could be experimentally and predictably directed by the timing of the secondary event (color change). That is, if the color change appeared prior to the key-press, participants perceived the key-press to occur earlier, and if the color change appeared after the tone presentation, participants perceived the tone to appear later than these events actually occurred. Thus, by manipulating the timing of the color change, we predictably evoked reverse binding effects in a pattern exactly opposite to typical temporal binding results. Moreover, the timing of the color change slightly affected binding, with the typical binding range (0–400 ms) after the event leading to slightly higher action binding effects. However, this effect is small and not very robust, and has therefore to be interpreted with caution.

An interesting result pattern can also be found in the novel control task *Baseline Memory*, which controlled the additional color identification, memorization, and reporting effort participants undertook in the baseline attention condition. Raw estimates show here that effects were estimated to occur significantly later than in the classic baseline condition, possibly indicating that the memorization and color report could additionally distract attentional resources towards the end of the trial. As such processes should also be present in the novel, experimental baseline attention condition, this emphasizes the need to control for these additional processes via a respective control (i.e., baseline memory condition) as we have done in the subsequent calculations for comparisons of the classic operant and novel baseline attention conditions.

While our result pattern supports our hypotheses, the question remains whether our interpretation of skewed attentional resources causing misestimations can be a valid explanatory alternative based on our knowledge of attentional processes. For this to be true, we have to assume that (1) attentional resources over time are limited, (2) attentional resources are malleable enough to adapt to contextual features, and (3) actions can draw these attentional resources similarly to external events. Indeed, attentional resources are assumed to be limited in general (Kahneman, 1973), and that holds also true for attentional processes sustained in time (e.g., Thomson et al., 2015). This automatically suggests that a broader time span for these attentional resources leads to less resources available for specific tasks and thus to performance decrements, or even to impaired perceptual sensitivity (Ling & Carrasco, 2006; Thomson et al., 2015). Although most studies on sustained attention focus on visual attention, there is evidence that visual and auditory sustained attention follow similar principles (Terashima et al., 2021). Moreover, for visual attention, evidence suggests attentional breadth to be extraordinarily malleable and adaptable (e.g., Gable & Harmon-Jones, 2010; Golubickis & Macrae, 2021; Goodhew & Plummer, 2019). Controlled actions especially seem to draw attentional processes easily, in spatial as well as temporal dimensions (e.g., Kirsch & Kunde, 2021; Schaaf et al., 2022; Schneider & Shiffrin, 1977; Wirth et al., 2018), and indeed attention seems an important factor in establishing metacognitive phenomena accompanying actions as well, such as the sense of agency (Cheyne et al., 2009; Hon, 2017). In further support of our interpretation, a recent review article also focuses on attentional processes as a defining aspect of temporal binding (Hon, 2022). Thus, current evidence and theories agree with our interpretation that the binding effects we demonstrated in all two-event sequences, be they action-effect sequences (*Operant Classic* task) or two-stimulus sequences (*Baseline Attention* task), compared to single events (*Baseline Classic* or *Baseline Memory* task), may be due to changes in attentional focus across time.

Importantly, attentional resource allocation seems to depend on sensory modality: if two events within a short time frame both rely on the same sensory modality, the same attentional resource is shared between both events, whereas different sensory modalities might partly rely on different attentional resources, at least for basic perceptual tasks (e.g., Arrighi et al., 2011; Keitel et al., 2013). This indicates that binding effects might increase if the main event and the distracting event share sensory modality and attentional resources.

Finally, we have to wonder if temporal binding – given its sensitivity to attentional distraction – can actually occur in most real-life settings. This question would present an exciting avenue for future studies in our view, possibly introducing tertiary, attention-capturing events with more or less saliency into classical operant action-effect sequences, thereby studying a possibly more ecologically valid scenario as the simple, distraction-free action-effect sequences we typically employ in the laboratory. For example, temporal binding effects for action-effect sequences with more than two events indicate that binding can occur for every event within that sequence dependent on the interval length between events, although action binding remains notoriously small or even absent (especially for middle events) in multiple-event sequences (Muth et al., 2022; Ruess et al., 2018). This replicates previous findings that the time perception of action events is not as malleable as the time perception of these actions’ effects, possibly due to the participants’ perceptual certainty regarding their own actions. Another layer to this explanation may be that action execution grabs more attentional resources than the action’s effect, thus directing the attentional focus skewed towards the action side of the action-effect sequence, further increasing perceptual certainty of actions compared to effects (see *Potential mechanisms* section). These findings suggest that even with tertiary attention-grabbing events interfering with typical action-effect sequences, binding might be found for all events; however, binding effects might possibly be skewed towards the event most likely to capture attentional resources.

### Potential mechanisms

The goal of this study was to identify and investigate attentional distraction as a potentially powerful methodological confound underlying temporal binding effects in Libet clock paradigms. While our results demonstrate that such an alternative explanation seems viable, at this point we cannot make clear inferences about the mechanisms underlying these processes. However, there are at least two clear potential candidates for mediating the effect of attention on temporal binding: partial multisensory integration (e.g., Debats et al., 2017; Klaffehn et al., 2021) and heuristic judgments (Reddy, 2021).

The multisensory integration account stipulates that individual sensory signals of a multimodal event may affect the perception of that multimodal event. The perceptual certainty of these individual sensory signals is thought to influence directly how much perceptive weight is given to any sensory signal in this multisensory integration process. That is, if perceptual certainty of one signal is higher than the other, the event with less perceptual certainty would be bound more strongly to the event with more perceptual certainty (see Fig. 1 of Tonn et al., 2021). This is also one explanation for stronger effect binding compared to action binding: perceptual certainty for the timing of the action execution is higher than for the timing of the effects of that action (see Fig. 1 of this article). It is plausible to assume that the quantity of attentional resources that may focus on any one event may directly influence the perceptual certainty regarding this event. That is, if attentional resources are drawn away from one event because they are (still or already) occupied by the second event in a two-event sequence, perceptual certainty of that one event should decrease and binding should accordingly increase, mirroring the results of the present study.

Likewise, it may be possible that due to attentional distraction, perceptual certainty of any event becomes so low that judgment heuristics are used instead for time estimations, favoring a “middle” ground in between the two events (Reddy, 2021). Future studies may probe for any of these mechanisms for temporal binding in two-event sequences.

### Limitations

An alternative explanation for our results lies with the fact that the color task in the attention condition (BA) focuses the participants’ attention on the clock itself, thereby potentially inviting participants to remember the timing of the color change and confusing or intermixing this time with the timing of the actual event they are supposed to monitor. We argue that, due to the nature of the task, the participants’ focus should be on the clock in any condition. In fact, we chose to localize the color change on the clock to avoid a third attention focus, in addition to the clock and the event the participants are asked to monitor, which would in itself constitute a confound. We thus believe that the influence of the same localization should not be too strong; nevertheless, it is a viable possibility that we cannot preclude on the basis of the current experiment.

Our study also does not allow us to make definitive statements about the necessity of causality between events or not. Although we took care to diminish the influence of causal perceptions on time estimations, we cannot preclude that participants perceived some sort of causality between color change and the event they were asked to evaluate in time, as color change was somewhat temporarily close to the key-press or tone in at least half of the trials. Interestingly, the random presentation of the color change (before vs. after the event) as well as binding effects even for color changes that occurred long after the respective event (see Fig. 4) suggest that causality is likely not critical for binding effects. Nevertheless, this association needs to be further investigated: can any secondary attention-capturing event direct binding scores irrespective of any causal relationship between the primary and secondary event?

Additionally, we want to stress that while our new experimental tasks employed a secondary attention-capturing event, we do not claim that the attention elicited for this secondary event is the same as would be present in action-effect sequences. For one, our secondary event was also relevant to the participants, and thus attention was deliberately focused on this task in contrast to the operant classic task in which the secondary event was irrelevant. However, previous studies indicate that action-effect sequences grab attention so reliably that we cannot help but monitor even irrelevant aspects of this sequence such as irrelevant action effects (e.g., Schaaf et al., 2022). That is, while the attentional processes at play may differ between the two tasks in this experiment, the fact that both events in both tasks may grab attentional resources is well established. While the main research question of the present study (“Is temporal binding a measure of skewed time perception due to acting or can it be elicited by any attention-grabbing two-event sequence, irrespective of action-effect processes?”) is not strongly affected by this potential difference, the question about which type of attention is needed for temporal binding to occur seems a fruitful avenue for future studies.

Finally, we do not want to make the claim that attentional resources are the only source for binding effects. In fact, there are several situational aspects that may affect the strength of temporal binding; likewise, there are situational contexts that may modulate the quantity of attentional resources needed for task completion. Such situational aspects include practice with the task, predictability of any events within the two-event sequence, or the participants’ pre-experimental biases and expectations (e.g., Hon, 2022; Isham & Wall, 2022; Matute et al., 2017; Ruess et al., 2017).

## Conclusions

In the present study, we identified and studied a profound methodological confound in typical temporal binding paradigms: the impact of a redirection of attentional resources in two-event sequences (as in classic operant conditions) versus singular events (as in classic baseline conditions). Our results indicate that binding effects in Libet clock paradigms may be based to a large degree on such attentional processes, irrespective of intention or action-effect sequences.

## Footnotes

1 Please note that while Haggard in his original study on temporal binding compared voluntary and involuntary movements (Haggard et al., 2002), some of his own subsequent work follows the experimental logic as explained here (e.g., Wenke et al., 2009), as well as countless other studies in the field (e.g., Antusch et al., 2021; Ruess et al., 2017; Saito et al., 2015; Schwarz et al., 2019a; Siebertz & Jansen, 2022).

2 Please note that we chose 400 ms as a threshold instead of 300 ms as some temporal binding effects within comparable action-effect sequences in Libet clock paradigms can still be found within the 400 ms interval (although relatively small; Haggard et al., 2002; Ruess et al., 2017), and we wanted to ensure that our comparison of a typical versus atypical time range was sound and not driven by artefacts introduced by a too short time frame in the atypical condition.

3 We use the term “effects” for tones in all conditions to adhere to common terminology within temporal binding research, although tones were not preceded by key-presses in all baseline conditions and are, thus, technically not effects.

## References

[CR1] Antusch S, Aarts H, Custers R (2019). The role of intentional strength in shaping the sense of agency. Frontiers in Psychology.

[CR2] Antusch S, Custers R, Marien H, Aarts H (2020). Intentionality and temporal binding: Do causality beliefs increase the perceived temporal attraction between events?. Consciousness and Cognition.

[CR3] Antusch S, Custers R, Marien H, Aarts H (2021). Intentional action and limitation of personal autonomy. Do restrictions of action selection decrease the sense of agency?. Consciousness and Cognition.

[CR4] Arrighi R, Lunardi R, Burr D (2011). Vision and audition do not share attentional resources in sustained tasks. Frontiers in Psychology.

[CR5] Cheyne JA, Carriere JS, Smilek D (2009). Absent minds and absent agents: Attention-lapse induced alienation of agency. Consciousness and Cognition.

[CR6] Debats NB, Ernst MO, Heuer H (2017). Perceptual attraction in tool use: evidence for a reliability-based weighting mechanism. Journal of Neurophysiology.

[CR7] Dewey JA, Knoblich G (2014). Do implicit and explicit measures of the sense of agency measure the same thing?. PLoS One.

[CR8] Gable P, Harmon-Jones E (2010). The blues broaden, but the nasty narrows: Attentional consequences of negative affects low and high in motivational intensity. Psychological Science.

[CR9] Golubickis M, Macrae CN (2021). That’s me in the spotlight: Self-relevance modulates attentional breadth. Psychonomic Bulletin & Review.

[CR10] Goodhew SC, Plummer AS (2019). Flexibility in resizing attentional breadth: Asymmetrical versus symmetrical attentional contraction and expansion costs depends on context. Quarterly Journal of Experimental Psychology.

[CR11] Haggard P (2017). Sense of agency in the human brain. Nature Reviews Neuroscience.

[CR12] Haggard P, Tsakiris M (2009). The experience of agency: Feelings, judgments, and responsibility. Current Directions in Psychological Science.

[CR13] Haggard P, Clark S, Kalogeras J (2002). Voluntary action and conscious awareness. Nature Neuroscience.

[CR14] Hoerl C, Lorimer S, McCormack T, Lagnado DA, Blakey E, Tecwyn EC, Buehner MJ (2020). Temporal binding, causation, and agency: Developing a new theoretical framework. Cognitive Science.

[CR15] Hon N (2017). Attention and the sense of agency: A review and some thoughts on the matter. Consciousness and Cognition.

[CR16] Hon, N. (2022). Attention and expectation likely underlie temporal binding measured using the Libet clock. *Quarterly Journal of Experimental Psychology*. 10.1177/17470218221132762 10.1177/17470218221132762 36214087

[CR17] Humphreys GR, Buehner MJ (2009). Magnitude estimation reveals temporal binding at super-second intervals. Journal of Experimental Psychology: Human Perception and Performance.

[CR18] Imaizumi S, Tanno Y (2019). Intentional binding coincides with explicit sense of agency. Consciousness and Cognition.

[CR19] Isham EA, Wall TA (2022). Differentiating the reported time of intent and action on the basis of temporal binding behaviors and confidence ratings. Attention, Perception, & Psychophysics.

[CR20] Ivanof BE, Terhune DB, Coyle D, Gottero M, Moore JW (2021). Examining the effect of Libet clock stimulus parameters on temporal binding. Psychological Research.

[CR21] Kahneman D (1973). *Attention and effort*.

[CR22] Keitel C, Maess B, Schröger E, Müller MM (2013). Early visual and auditory processing rely on modality-specific attentional resources. Neuroimage.

[CR23] Kirsch W, Kunde W (2021). The size of attentional focus modulates the perception of object location. Vision Research.

[CR24] Kirsch W, Kunde W, Herbort O (2019). Intentional binding is unrelated to action intention. Journal of Experimental Psychology: Human Perception and Performance.

[CR25] Klaffehn AL, Sellmann FB, Kirsch W, Kunde W, Pfister R (2021). Temporal binding as multisensory integration: Manipulating perceptual certainty of actions and their effects. Attention, Perception, & Psychophysics.

[CR26] Kok A, Ridderinkhof KR, Ullsperger M (2006). The control of attention and actions: current research and future developments. Brain Research.

[CR27] Libet B, Gleason CA, Wright EW, Pearl DK (1983). Time of conscious intention to act in relation to onset of cerebral activity (readiness-potential): The unconscious initiation of a freely voluntary act. Brain.

[CR28] Ling S, Carrasco M (2006). When sustained attention impairs perception. Nature Neuroscience.

[CR29] Loftus GR, Masson ME (1994). Using confidence intervals in within-subject designs. Psychonomic Bulletin & Review.

[CR30] Majchrowicz B, Wierzchoń M (2018). Unexpected action outcomes produce enhanced temporal binding but diminished judgement of agency. Consciousness and Cognition.

[CR31] Matute H, Cubillas CP, Garaizar P (2017). Learning to infer the time of our actions and decisions from their consequences. Consciousness and Cognition.

[CR32] Moore JW (2016). What is the sense of agency and why does it matter?. Frontiers in Psychology.

[CR33] Moore JW, Ruge D, Wenke D, Rothwell J, Haggard P (2010). Disrupting the experience of control in the human brain: pre-supplementary motor area contributes to the sense of agency. Proceedings of the Royal Society B: Biological Sciences.

[CR34] Muth FV, Wirth R, Kunde W (2022). Temporal binding in multi-step action-event sequences is driven by altered effect perception. Consciousness and Cognition.

[CR35] Pfister R, Janczyk M (2013). Confidence intervals for two sample means: Calculation, interpretation, and a few simple rules. Advances in Cognitive Psychology.

[CR36] Pfister R, Tonn S, Weller L, Kunde W, Schwarz KA (2021). To prevent means to know: Explicit but no implicit agency for prevention behavior. Cognition.

[CR37] Reddy NN (2021). The implicit sense of agency is not a perceptual effect but is a judgment effect. Cognitive Processing.

[CR38] Ruess M, Thomaschke R, Kiesel A (2017). The time course of intentional binding. Attention, Perception, & Psychophysics.

[CR39] Ruess M, Thomaschke R, Haering C, Wenke D, Kiesel A (2018). Intentional binding of two effects. Psychological Research.

[CR40] Saito N, Takahata K, Murai T, Takahashi H (2015). Discrepancy between explicit judgement of agency and implicit feeling of agency: Implications for sense of agency and its disorders. Consciousness and Cognition.

[CR41] Schaaf M, Kunde W, Wirth R (2022). Monitoring goal-irrelevant effects interferes with concurrent tasks. Acta Psychologica.

[CR42] Schneider W, Shiffrin RM (1977). Controlled and automatic human information processing: I. Detection, search, and attention. Psychological Review.

[CR43] Schwarz, K. A., Weller, L., Klaffehn, A. L., & Pfister, R. (2019a, a). The effects of action choice on temporal binding, agency ratings, and their correlation. *Consciousness and Cognition, 75,* 102807. 10.1016/j.concog.2019.102807 31494358

[CR44] Schwarz, K. A., Weller, L., Pfister, R., & Kunde, W. (2019b, b). Connecting action control and agency: Does action-effect binding affect temporal binding?. *Consciousness and Cognition, 76,* 102833. 10.1016/j.concog.2019.102833 31629097

[CR45] Siebertz M, Jansen P (2022). Diverging implicit measurement of sense of agency using interval estimation and Libet clock. Consciousness and Cognition.

[CR46] Suzuki K, Lush P, Seth AK, Roseboom W (2019). Intentional binding without intentional action. Psychological Science.

[CR47] Synofzik M, Vosgerau G, Voss M (2013). The experience of agency: an interplay between prediction and postdiction. Frontiers in Psychology.

[CR48] Tanaka T, Matsumoto T, Hayashi S, Takagi S, Kawabata H (2019). What makes action and outcome temporally close to each other: A systematic review and meta-analysis of temporal binding. Timing & Time Perception.

[CR49] Terashima H, Kihara K, Kawahara JI, Kondo HM (2021). Common principles underlie the fluctuation of auditory and visual sustained attention. Quarterly Journal of Experimental Psychology.

[CR50] Thomson DR, Besner D, Smilek D (2015). A resource-control account of sustained attention: Evidence from mind-wandering and vigilance paradigms. Perspectives on Psychological Science.

[CR51] Tonn S, Pfister R, Klaffehn AL, Weller L, Schwarz KA (2021). Two faces of temporal binding: Action-and effect-binding are not correlated. Consciousness and Cognition.

[CR52] Tramacere A, Allen C (2022). Temporal binding: digging into animal minds through time perception. Synthese.

[CR53] Venskus A, Ferri F, Migliorati D, Spadone S, Costantini M, Hughes G (2021). Temporal binding window and sense of agency are related processes modifiable via occipital tACS. PLoS One.

[CR54] Weller L, Schwarz KA, Kunde W, Pfister R (2020). Something from nothing: Agency for deliberate nonactions. Cognition.

[CR55] Wenke D, Waszak F, Haggard P (2009). Action selection and action awareness. Psychological Research.

[CR56] Wirth R, Janczyk M, Kunde W (2018). Effect monitoring in dual-task performance. Journal of Experimental Psychology: Learning, Memory, and Cognition.

